# A rapamycin derivative, biolimus, preferentially activates autophagy in vascular smooth muscle cells

**DOI:** 10.1038/s41598-018-34877-8

**Published:** 2018-11-08

**Authors:** Yerin Kim, Jun Kyu Park, Jun-Hyuk Seo, Hyun-Seung Ryu, Kyung Seob Lim, Myung Ho Jeong, Dong Hoon Kang, Sang Won Kang

**Affiliations:** 1 Department of Life Science, Ewha Womans University, Seoul, 03760 Republic of Korea; 2 Vasthera Co. Ltd, Seoul, 03760 Republic of Korea; 3 CGBio Ltd, Jangseong, 57248 Republic of Korea; 4 Cardiovascular Research Center, Chonnam National University, Gwangju, 61469 Republic of Korea; 5 Present Address: Department of Asan Institute for Life Science, Asan Medical Center, College of Medicine, University of Ulsan, Seoul, 05505 Republic of Korea

## Abstract

Although rapamycin is a well-known conformational inhibitor of mTORC1, it is now widely used for treating arterial restenosis. Various rapamycin analogues (rapalogue) have been made for applying to drug-eluting stents. Here we show that two major rapalogues, everolimus and biolimus, exert a differential effect on the mTORC1-mediated signaling pathways in vascular smooth muscle cells. In balloon-injured carotid arteries, both rapalogues strongly inhibit neointimal hyperplasia. Signaling pathway analyses reveal that everolimus exert cytotoxicity by increasing cellular reactive oxygen species and consequently reduce energy metabolism. By contrast, biolimus confers a preferential induction of autophagy by more strongly activating major autophagy regulator, ULK1, in vascular smooth muscle cells than everolimus does. As a consequence, the implantation of biolimus-eluting stent reduces endothelial loss, which in turn reduces inflammation, in porcine coronary arteries. Thus, this study reveals that a chemical derivatization can cause a change among mTORC1-dependent signaling pathways in vascular smooth muscle cells, thereby enabling to elicit a differential efficacy on arterial restenosis.

## Introduction

Balloon angioplasty and stent implantation have been applied to extend arterial vessels narrowed by atherosclerosis^1^. However, through the procedure, endothelial cells are denuded and thus the underlying vascular smooth muscle cells (VSMC) are exposed to blood stream containing macrophage/monocytes. As a result, VSMCs de-differentiate and aggressively proliferate, thereby causing in-stent restenosis^2^. To prevent such complications, drug-eluting stents coated with cytostatic/cytotoxic drugs that inhibit VSMC proliferation have been developed and widely used in clinics^3^. Nonetheless, those drugs also suppress re-endothelialization and thus induce inflammation and thrombosis.

Rapamycin is one of the most widely used drugs for drug-eluting stent^4^. Rapamycin was identified from soil bacterium *S. hygroscopicus* and it was initially known for its broad immunosuppressive and anti-proliferative effects^5,6^. Later, it was found that TOR protein (mTOR in mammals) is the target of rapamycin^7,8^. mTOR, a Ser/Thr protein kinase with many substrates, is a central regulator of cell proliferation, metabolism, and autophagy^9,10^. The mTOR kinase forms functionally distinct two complexes, mTORC1 and mTORC2, of which only mTORC1 is sensitively suppressed by rapamycin^10^. In mammalian cells, rapamycin firstly binds to a FKBP12 protein and then this complex allosterically inhibits mTORC1 by binding to the FRB domain next to the kinase domain of mTOR^11^. Recent studies have shown that some mTORC1 substrates are resistant to rapamycin in several cell types^12,13^. The p70S6K, a mTORC1 substrate involved in protein translation^14^, is fully inhibited by rapamycin in most cell types, while the multiple phosphorylation sites^15^ of another protein translation regulator 4E-BP1^16^ show a differential degree of de-phosphorylation responding to the rapamycin-induced mTORC1 inhibition^13^. Also, the mTORC1-dependent phosphorylation of autophagy-inducing kinase ULK1 at S758^17,18^ is resistant to rapamycin^12^.

To date, many rapamycin analogues (rapalogue) have been developed to improve drug delivery and lower toxicity^19^. In general, rapamycin was broadly modified on C40 branch that does not bind to either the FRB domain of mTOR or the FKBP12 protein^11^. Those analogues exhibit different bioavailability and stability *in vivo*^20^. For example, the natural rapamycin sirolimus has a disadvantage of causing thrombosis^4,21^, whereas everolimus prevents such in-stent thrombosis^22^. The latest version of rapalogues is biolimus that shows an improved drug delivery capacity as it has more lipophilic properties^23^. Nonetheless, the differential signaling impact among rapalogues has not been evaluated seriously in VSMCs.

In this study, we found that biolimus is unexpectedly preferential to autophagy induction by enhancing the dephosphorylation of ULK at S758. Mechanistically, sirolimus and everolimus were cytotoxic by inducing cell death, while biolimus was rather cytostatic by inducing autophagy. More importantly, biolimus-eluting stents lowered inflammation score when it was implanted in porcine coronary arteries. Overall, our signaling study implicates the importance of structure-activity relationship for drug derivatization.

## Materials and Methods

### Reagents

Sirolimus (Cat No. 37094), everolimus (Cat No. 07741), anti-LC3B antibody (Cat No. L7543) were purchased from Sigma-Aldrich. Biolimus (Cat No. sc-391515) and anti-pRb antibody (Cat No. sc-50) were purchased from Santa Cruz Biotechnology. Anti-p-mTOR (pS2448) (Cat No. 5536 S), Anti-mTOR (Cat No. 2972 S), Anti-p-ULK1 (pS758) (Cat No. 14202 S), Anti-ULK1 (Cat No. 6439 S), Anti-p-4EBP1 (pS65) (Cat No. 9451 S), Anti-4EBP1 (Cat No. 9644), Anti-p-P70S6K (pT389) (Cat No. 9205 S), Anti-P70S6K (Cat No. 9202 S) antibodies were purchased from Cell Signaling Technology. Anti-Ki67 antibody (Cat No. 550609) was purchased from BD Pharmingen. Alexa Fluor 488 (Cat No. A21206) and 568-conjugated donkey anti-rabbit secondary antibodies (Cat No. A10042) and Alexa Fluor 568-conjugated donkey anti-mouse secondary antibodies (Cat No. A10037) were purchased from Invitrogen. pRb (pThr373) antibody (Cat No. 9306 S) was purchased from New England Biolabs.

### Cell culture

Human aortic SMCs (HASMC) were purchased from Lonza. Cells were cultured using Smooth Muscle Cell Growth Medium (SmGM) containing 5% fetal bovine serum with growth factors and antibiotics (Lonza, Cat no. cc-4149) in humidified incubator containing 5% CO_2_ at 37 °C. All experiments were performed with HASMCs at passages 5–7.

### Immunoblot analysis

Cells were rinsed twice with ice-cold phosphate-buffered saline (PBS) and were frozen on liquid nitrogen. Then, they were lysed in a lysis buffer containing 20 mM HEPES (pH 7.0), 1% Triton X-100, 150 mM NaCl, 10% glycerol, 1 mM EDTA (pH 8.0), 2 mM EGTA (pH 8.0), 1 mM DTT, 5 mM Na_3_VO_4_, 5 mM NaF, 1 mM AEBSF, 5 μg/ml aprotinin, and 5 μg/ml leupeptin. The cell lysates were centrifuged at 15,000 ×g for 15 min, and the protein concentrations were determined by Bradford assay (Pierce). Protein samples were mixed with SDS sample buffer and boiled for 5 min. The proteins were separated by SDS-PAGE and transferred onto nitrocellulose membranes by electroblotting for 1 hr. The membranes were blocked with 5% bovine serum albumin (BSA) or 5% dry skimmed milk in Tris-buffered saline containing 0.05% (v/v) Tween-20 (TBST) for 2 hr ar room temperature and then incubated with the appropriate primary antibody (1:1,000 dilution) in blocking buffer at 4 °C overnight. After washing three times with TBST, membranes were incubated with horseradish peroxidase-conjugated secondary antibody (Amersham Biosciences) in blocking buffer. The immune-reactive bands were detected with an enhanced chemiluminescence kit (AbFrontier, Korea) and quantified by a LAS-3000 imaging system (Fuji Film, Japan). When necessary, the membranes were stripped by shaking them for 60 mins at 37 °C in 67 mM Tris (pH 6.7), 2% SDS, and 100 mM β-mercaptoethanol and reprobed with the appropriate pan-antibody.

### Cell viability assay

HASMCs (3,000 cells) were seeded in 100 μl of complete media on white flat bottom 96-well plate (3917, Corning Costar). After 24 hr, rapalogues in ethanol were treated to cells at indicated concentrations and incubated for additional 24 hr. Cellular ATP concentration was measured using CellTiter-Glo Luminescent Assays kit (Promega) according to manufacturer’s protocol. Luminescence was measured by MicroLumat Plus LB96V (Berthold Technologies, USA).

### Flow cytometry Analysis

HASMCs (1 × 10^5^ cells) were cultured on 35-mm tissue culture dish (153066, Nunc) for 24 hr and then treated with rapalogues for additional 24 hr. For cell cycle analysis, cells were detached and permeabilized in 70% ethanol for overnight at −20 °C. The cells were treated with RNase A (100 μg/ml in PBS) for 1 hr at 37 °C and then stained with propidium iodide (final conc. 10 μg/ml).

For determination of S-phase cells, cells were incubated with 10 μM BrdU for 2 hr at 37 °C and then analyzed using BrdU Flow Kits (BD Biosciences) according to manufacturer’s protocol. Briefly, cells were fixed and permeabilized, and incubated with 300 μg/ml of DNase for 1 hr at 37 °C. FITC-conjugated anti-BrdU antibody was incubated with cells for 20 min at room temperature in the dark. Then, 7-AAD was treated just before analysis for total DNA contents. The percentages of G_0_/G_1_ diploid cells were analyzed by Modfit LT software (Verity Software House, Topsham, ME) in FACSCalibur system (BD biosciences). The percentages of BrdU-positive cells were analyzed by FlowJo software (Tree Star).

### Balloon-induced injury procedure in rat carotid artery

Animal experiments were approved by the Institutional Animal Care and Use Committee (IACUC) of Ewha Womans University and conformed to *Guide for Care and Use of Laboratory Animals* published by the US National Institutes of Health (The National Academies Press, 8^th^ Edition, 2011). The ten-week-old male Sprague-Dawley rats were used for a balloon-induced injury model. A balloon injury was created using an infiltrated 2 F Fogarty balloon embolectomy catheter in the left common carotid artery as previously described^12^. In brief, the rats were anaesthetized by inhalation of isoflurane gas (N_2_O: O_2_ / 70%: 30%). Then, the left external carotid artery was exposed and arterial branches were electro-coagulated. A catheter was inserted through the transverse arteriotomy of the external carotid artery and positioned in 1 cm distance from a cut-down site. The catheter was then inflated and moved back and forth three times along the common carotid artery. For catheter-mediated intramural delivery of drugs^24^, the solutions of rapalogues (20 μM in 200 μl of PBS) were infused into the injured carotid lumen and then incubated for 15 min to allow the efficient drug diffusion. The lumen was flushed once with saline before the catheter was removed. After the removal of catheter, the punched area was sealed and the clap in the common carotid artery was released to reestablish blood flow. Unless otherwise stated, the rats were then recovered in the cages for 10 days.

### Immunofluorescence staining

Paraffin sections of balloon injured carotid Arteries were de-paraffinized in xylene and rehydrated in ethanol. The rehydrated tissue sections were boiled for 20 min in citric acid-based antigen unmasking solution (Vector Laboratories) for antigen retrieval and then blocked with 5% normal donkey serum in PBST (0.3% Triton X-100 in PBS) for 1 hr. Then, the samples were incubated with anti-LC3B antibody (1: 200 dilution) or anti-Ki67 antibody (1: 50 dilution) overnight at 4 °C. After washing with PBST three times, the samples were incubated with Alexa Fluor 488 - or 568 - conjugated donkey anti-rabbit IgG antibodies for LC3B and Alexa Fluor 568 - conjugated donkey anti-mouse IgG antibodies for Ki67 for 2 hr at room temperature in the dark. Nuclear DNA was labeled with DAPI. Fluorescence image were obtained using LSM 510 META confocal microscope (Carl Zeiss).

### TUNEL assay

De-paraffinized, rehydrated and antigen retrieved paraffin sections were incubated with TUNEL reaction mixture for 60 min at 37 °C using *In Situ* Cell Death Detection Kit (Roche Diagnostics), according to the manufacturer’s instructions. Nuclear DNA was labeled with DAPI.

### Purification and quantification of rapalogues

HASMCs (8 × 10^5^ cells) were seeded on 60 - mm tissue culture dishes (245389, Nunc). After 24 hr, rapalogues in ethanol were treated for 2 hr to the cells at final concentrations of 2 μM or 10 μM in 2.5 ml culture media. The cells washed with PBS quickly and extracted with 1 ml methanol for 2 hr at −20 °C. After centrifugation at 12,000 × g for 10 min, Supernatants were dried using a savant SPD121P Speedvac Concentrator (Thermo Fisher Scientific), dissolved in the 200 μl of 50% Acetonitrile with 0.1% trifluoroacetic acid solution. Samples were separated by a reverse-phase high performance liquid chromatography (Agilent Series 1100 HPLC system with diode array). 100 μl of each samples were injected to a C_18_ column (4.6 × 250 mm ID, 5 μm spherical packing; Vydac) and eluted through 50–95% Acetonitrile with 0.1% trifluoroacetic acid mobile phase solution at a rate of 1.2 ml/min. Amounts of extracted drugs from cells were calculated by using each standard curves gained from standard drug solutions (1.25, 2.5, 5, 10, 20 μM). Drugs were detected by UV absorbance at 278 nm.

### Coating of drug on metal stent

Coating method was used by an ultrasonic spray method as described previously^25^. Briefly, 20 mg of sirolimus, evolimus, or biolimus with 20 mg of poly lactic acid (PLA, 0.80 ~1.2 dL/g of inherent viscosity in chloroform at 0.1 w/v% at 25 °C) were dissolved in 5 mL of tetrahydrofuran (THF), respectively. The coating solutions were spray-coated on the metal stent (SoniCoater, Noanix Co., Korea) and then were vacuum-dried for 24 hr. Coating thickness measured by reflection spectrometry (F40, Filmetrics, Inc., USA) was 6.5 ± 2.46 μm.

### Stent implantation procedure

The animal study was approved by the Ethics Committee of Chonnam National University Medical School and Chonnam National University Hospital, and conformed to *Guide for Care and Use of Laboratory Animals* published by the US National Institutes of Health (The National Academies Press, 8^th^ Edition, 2011). The mini-pigs weighing 25–30 kg were anesthetized with zolazepam and tiletamine (2.5 mg/kg, Zoletil50, Virvac, Caros, France), xylazine (3 mg/kg, Rompun, Bayer AG, Leverkusen, Germany) and azaperone (6 mg/kg, Stresnil, Janssen-Cilag, Neuss, Germany). They received supplemental oxygen continuously through an oxygen mask. After subcutaneous administeration of 2% lidocaine at the cut-down site, the left carotid artery was surgically exposed. A 7 French (F) sheath was inserted. Also, heparin (5,000 U) was administered intravenously as a bolus prior to the procedure. The target coronary artery was engaged using standard 7 F guide catheters and control angiograms of both coronary arteries were performed using nonionic contrast agent in two orthogonal views. The stent was deployed by inflating the balloon and the resulting stent-to-artery ratio was 1.3:1. Coronary angiograms were obtained immediately after stent implantation. All equipment was removed and the carotid artery ligated. The bare metal stent (BMS) and drug coated BMS were implanted in the left or right femoral arteries alternatively in each pig. Continuous hemodynamic and surface electrocardiographic monitoring was maintained throughout the procedure. To prevent acute thrombosis after stenting, aspirin 100 mg and clopidogrel 75 mg were administered daily for 5 days before the procedure, which was continued until sacrifice stage. After day 28 post-implantation, stenting, the animals underwent follow-up angiography in the same orthogonal views before sacrifice with 5 ml of potassium chloride via the intracarotid artery. The part of the arteries surrounding the stent were removed carefully and processed for histological analysis.

### Histopathological analysis

The balloon-injured rats were anesthetized and the common carotid arteries were excised after transcardiac perfusion-fixation with heparinized saline containing 3.7% formaldehyde. The vessels were paraffin embedded and sectioned by rotary microtome (Leica RM2255). The two serial tissue sections (4 μm in thickness) were obtained from the middle area of common carotid arteries and stained with haematoxylin and eosin (HE). The luminal, internal elastic laminal, and external elastic laminal areas were measured using NIH Image v 1.62. The intimal and medial areas were determined by subtraction of the luminal area from the internal elastic area and by subtraction of the internal elastic area from the external elastic area. The values from two serial sections per rat were averaged for analysis.

The stented coronary arteries were dissected and histopathological analysis was performed by cardiovascular pathologists. The specimens were cut 50–100 µm in thickness, and stained with hematoxylin and eosin (HE). Quantitative measurements of the samples were performed using a calibrated microscope, digital video imaging system, and a microcomputer program (Visus 2000 Visual Image Analysis System, IMT Tech). Borders were manually traced for lumen area, area circumscribed by the internal elastic lamina, and the innermost border of the external elastic lamina (external elastic lamina area). Morphometric analysis of the neointimal area for a given vessel was calculated as the measured internal elastic lamina (IEL) area minus lumen area. The measurements were made on five cross-sections from the proximal, middle, and distal ends of each stented segment. Histopathological stenosis was calculated as 100 × (lesion neointimal area/lesion internal elastic lamina area). Injury, inflammation, and fibrin scores were determined by methods of Schwartz^26^, Kornowaski^27^, and Suzuki^28^, respectively. Ordinal data for the inflammation, fibrin, and endothelial scores were collected on each stent section using a scale of 0 to 3. The endothelial score for evaluation of artery healing was as follows: 0 = no generation, 1= <25%, 2 = 20~75%, 3= complete circumferential endothelialization.

### Statistical Analyses

All the graphs shows the mean values with error bars from three or more repeated experiments. Student’s *t*-test was performed to determine the statistical significance between two groups. One-way ANOVA with Turkey’s test was used to determine the overall statistical significance of three drug groups. Two-way ANOVA with Turkey’s test was used when concentration or time parameters were involved. A *P* < 0.05 was considered to be statistically significant.

## Results

### A rapamycin analogue biolimus exhibit a cytostatic, not cytotoxic, effects in VSMCs

**Figure 1:**
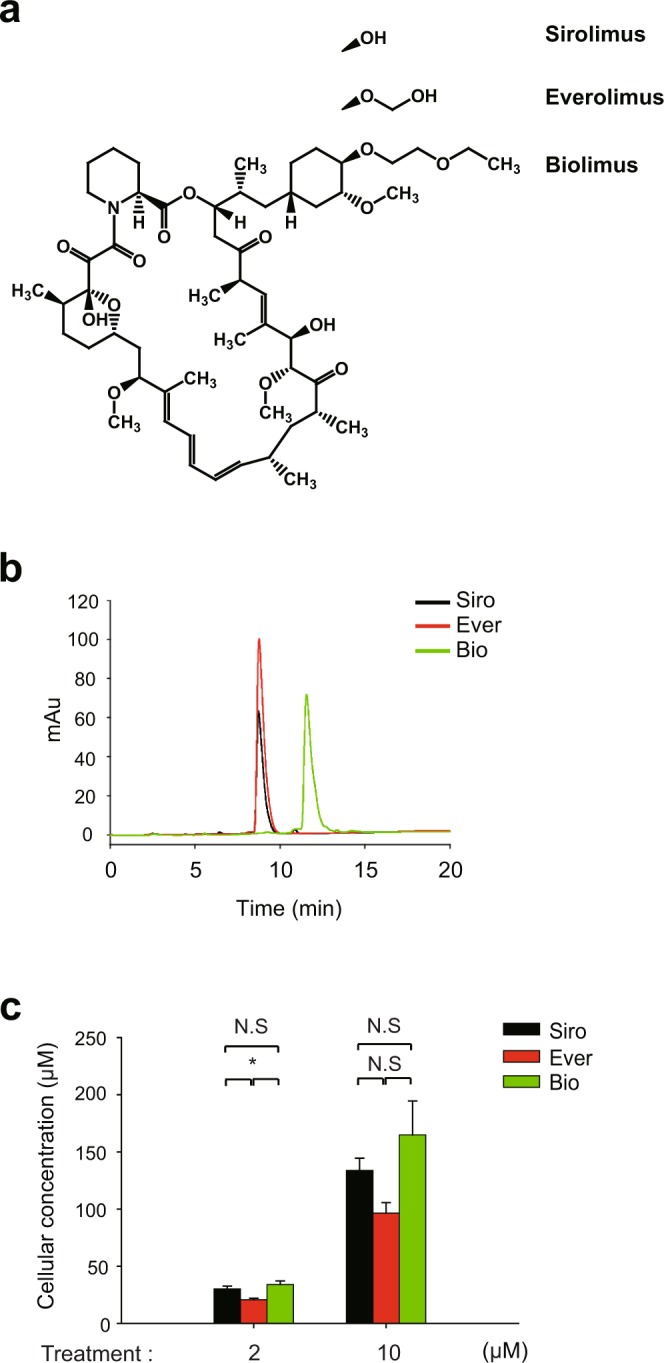
Cellular uptake of rapamycin analogues in VSMCs. (**a**) Chemical structures of three rapalogues- sirolimus (Siro), everolimus (Ever), and biolimus (Bio). Structures are drawn using ChemDraw software (PerkinElmer). Different moieties attached to C40 of the common macrolide core are shown. (**b**) Elution profile of rapalogues from high-performance liquid chromatography. The indicated rapalogue in methanol (10 μM) was separated on C_18_ column and then eluted by 50–95% acetonitrile gradient in 0.1% trifluoroacetic acid solution. The chromatogram shows the different retention times and absorbance intensities of the drugs measured at 278 nm. (**c**) Quantification of the concentration of rapalogue absorbed in HASMCs. The methanol extracts from cultured cells treated with rapalogues (2 and 10 μM in PBS) for 2 hr were separated on C_18_ column as in (B). Concentration was calculated from a standard curve of each compound. Bar in the graph are means ± SD of intracellular concentrations of rapalogues (*n* = 3, ^*^P < 0.05 with Student’s t-test). N.S., not significant.

To understand the molecular mechanism underlying the differential clinical outcome of rapalogues, we performed the signaling pathway analysis in VSMCs using two major rapalogues, everolimus, and biolimus. We also include the rapamycin (also known as sirolimus) as a standard drug. These drugs equally act as the conformational inhibitor of the mTORC1 complex^29^ but have different chemical moiety at C40 residue (Fig. 1a). Thus, we primarily tested the drug uptake by VSMCs^30^. When standard compounds were analyzed on C18 reverse-phase column by high-performance liquid chromatography, biolimus was eluted much later than other rapalogues (Fig. 1b), which confirmed that it is a more lipophilic version of rapamycin. To quantify the cellular uptake of drugs, VSMCs were incubated with drugs (2 or 10 μM in 2.5 ml culture media) for 2 hr and subjected to the methanol extraction. The methanol extracts of drug-treated VSMCs ran through C_18_ reverse-phase column for analysis. As a result, sirolimus and two other rapalogues showed a similar intracellular uptake in VSMCs (Fig. 1c). Although everolimus seemed to be slightly less permeable, the result indicated that cellular uptake does not matter to determine the efficacy of drugs tested in the study.

**Figure 2:**
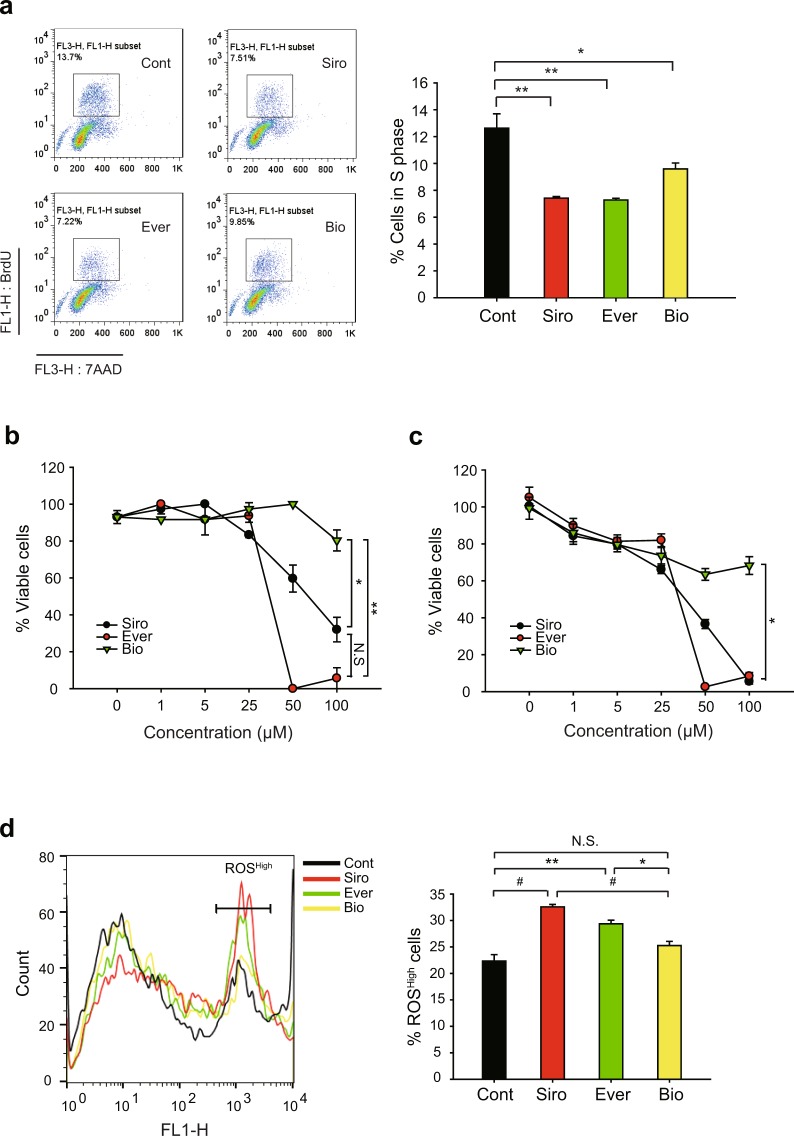
Comparisons of the effects of rapamycin analogues on HASMCs cell cycle progression and cell viability. (**a**) Cell cycle analysis of rapalogue-treated HASMCs. Cells were treated with 2 μM rapalogues for 24 hr and then labelled with BrdU and 7-AAD. The labelled cells were separated by fluorescence-assorted cell sorting (FACS). Representative FACS images including S-phase cells (*inner box*) are shown. Data in the graph show means ± SD of the percentage of BrdU^+^ cells (*n* = 3, *P < 0.05, ^**^P < 0.01 with Student’s t-test). (**b**,**c**) Cell viability was measured by trypan blue staining (**b**) and CellTiter-Glo reagent (**c**). Cells were treated with rapalogues at increasing concentrations for 24 hr. Cells were collected and either stained by trypan blue or incubated with CellTiter-Glo reagent. In latter assay, the ATP-based luminescent signal was measured by a luminomer. Data in the graph show means ± SD of the percentages of live cells versus total cells (*n* = 3, *P < 0.05, **P < 0.001 with two-way ANOVA). N.S., not significant. (**d**) Intracellular reactive oxygen species (ROS) level in rapalogue-treated HASMCs. Cells were treated with 50 μM rapalogues for 2 hr and then incubated with 3 μM CM-H_2_DCFDA for additional 30 min. The labelled cells were analyzed by FACS. Representative histogram, where cells with high ROS level are marked, is shown. Data in the graph show means ± SD of the percentage of ROS^High^ cells (*n* = 3, *P < 0.05, **P < 0.01, ^#^*P* < 0.005 with Student’s t-test). N.S., not significant.

Next, we analyzed cell cycle progression in drug-treated VSMCs. As expected, drug treatments similarly elicited cell cycle G1 arrest in proliferating VSMCs (Supplementary Figure S1a). Consistently, the BrdU incorporation assay also showed that, along with sirolimus, two rapalogues effectively reduced the cell number in S phase (Fig. 2a). To see the cell cycle arrest by rapalogues in a molecular level, we examined the inhibitory phosphorylation of pRb, a tumor suppressor that inhibits G1-S cell cycle progression^31,32^. The inhibitory phosphorylation on Thr373 residue was evidently reduced by the drug treatments (Supplementary Figure S1b). However, it was noted that biolimus was likely to be less effective on cell cycle arrest than the other drugs. To further see the cytotoxic effect of drugs tested, we examined the SMC viability following treatment with increasing drug concentrations. Sirolimus and everolimus induced severe cell death at higher concentrations relative to the concentrations eliciting cell cycle arrest (IC_50_ = 54.24 μM for sirolimus, 29.50 μM for everolimus) (Fig. 2b). In contrast, biolimus did not exert cytotoxicity to VSMCs even at the highest concentrations tested (up to 100 μM of treatment concentration). Consistent with the tryphan blue assay, the ATP-based viability assay also revealed that sirolimus and everolimus drastically induced a necrotic death above 25 μM, while biolimus still had a modest effect on it (Fig. 2c). Indeed, sirolimus and everolimus significantly increased intracellular level of reactive oxygen species (ROS) in VSMCs, whereas biolimus did not (Fig. 2d). Collectively, the data indicated that sirolimus and everolimus, not biolimus, preferentially induce cell death by reducing energy metabolism and consequent ROS production.

#### Biolimus preferentially induces autophagy

**Figure 3:**
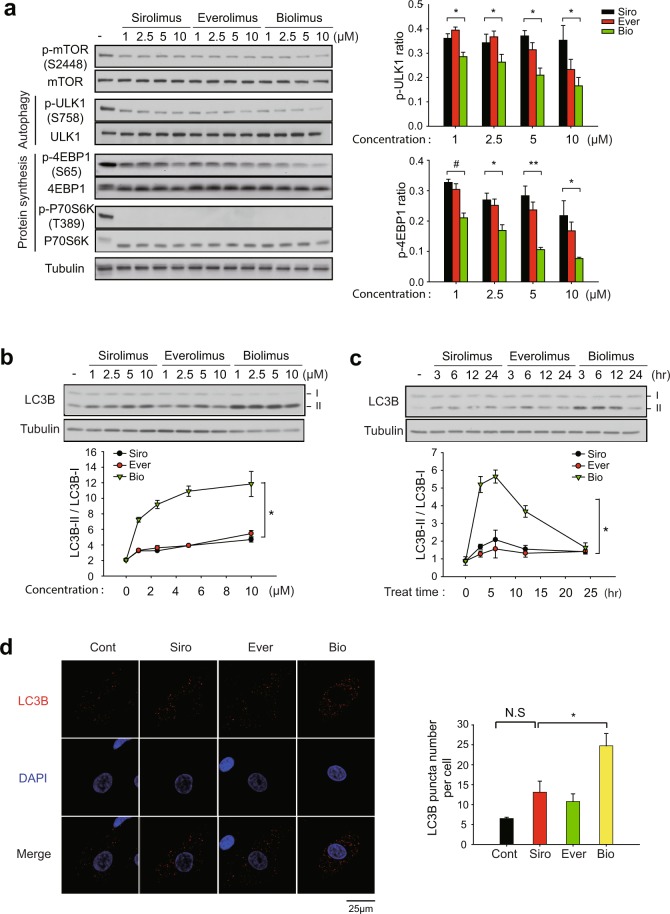
Differential effect of rapamycin analogues on mTORC1 substrates de-phosphorylation and autophagy induction. (**a**) Phosphorylation-specific immunoblot analysis of mTORC1 substrates in HASMCs. Cells were pretreated with rapalogues at increasing concentrations for 2 hr. The phospho-specific bands were quantified and normalized by the intensities of corresponding unphosphorylated protein bands. Data in graph show means ± SD of fold changes of ULK1 and 4EBP1 phosphorylation versus untreated control sample (*n* = 3, ^*^P < 0.05, ^**^P < 0.01, ^#^*P* < 0.005 with Student’s t-test). (**b**,**c**) Immunoblot analysis of LC3B conversion in the rapalogue-treated HASMCs. Cells were pretreated with rapalogues at increasing concentrations for 2 hr (**b**) or with 1 μM rapalogues for indicated times (**c**). Data in the graph show means ± SD of the ratio of LC3B-II versus LC3B-I (*n* = 3, ^*^P < 0.001 with two-way ANOVA). (**d**) Immunofluorescence staining of LC3B in the rapalogue-treated HASMCs. HASMCs were pretreated with 2 μM rapalogues for 3 hr. The number of LC3B punta was averaged from 40 cells per experiment. Data in the graph show means ± SEM of the number of LC3B puncta per cell (*n* = 3, ^*^P < 0.05 with Student’s t-test). N.S., not significant. Representative immunoblots and images are shown.

We postulated that biolimus may elicit cell cycle arrest through a distinct signaling pathway. Since the mTORC1 complex is involved in various signaling pathways for protein translation, metabolism, and autophagy^33^, we examined the activation level of key signaling molecules downstream of mTORC1 in the drug-treated VSMCs. Sirolimus and two rapalogues all similarly reduced the mTOR phosphorylation and completely inhibited the activation of p70S6K, a central regulator of protein synthesis, which might be the underlying mechanism for the similar effect on cell cycle arrest among drugs (Fig. 3a). It is noted that complete de-phosphorylation of Thr 389 residue on p60S6K was confirmed by electrophoretic mobility in total p70S6K blot^34^. However, the activation of another protein synthesis regulator 4E-BP1, represented by mTORC1-dependent phosphorylation at Ser65 residue, was more effectively inhibited by biolimus compared to sirolimus and everolimus. Moreover, the level of inhibitory phosphorylation at Ser758 site on ULK1 was also decreased to a greater extent by biolimus (Fig. 3a). Thus, the data suggested that biolimus is certainly distinct, while sirolimus and everolimus are very similar, in term of signaling pathway specificity.

Since the previous studies have emphasized the importance of autophagy in the vascular environment^35^, we focused the distinct effect of biolimus on ULK1 as a key autophagy inducer. Therefore, we examined the autophagy induction in the drug-treated VSMCs. LB3B is a key molecular marker for autophagosome formation because the cytosolic form of LC3B (LC3B-I) is converted to phosphatidylethanolamine conjugate (LC3B**-**II) recruited to autophagosomal membranes^36^. As a result, biolimus treatment preminently increased LC3B-II level in both a dose- and time-dependent manner compared to other drugs (Fig. 3b and c). In addition, level of LC3B-II was similar between sirolimus and everolimus treatments. Subsequently, we measured the number of autophagosomes by immunofluorescence staining of LC3B in VSMCs. Indeed, biolimus treatment markedly increased the number of LC3B-positive punctas compared to those in sirolimus and everolimus treatments (Fig. 3d). Overall, these results demonstrate that biolimus preferentially induces autophagy, thereby resulting in the inhibition of VSMC proliferation.

### Biolimus inhibits neointimal hyperplasia via autophagy induction in injured carotid arteries

**Figure 4:**
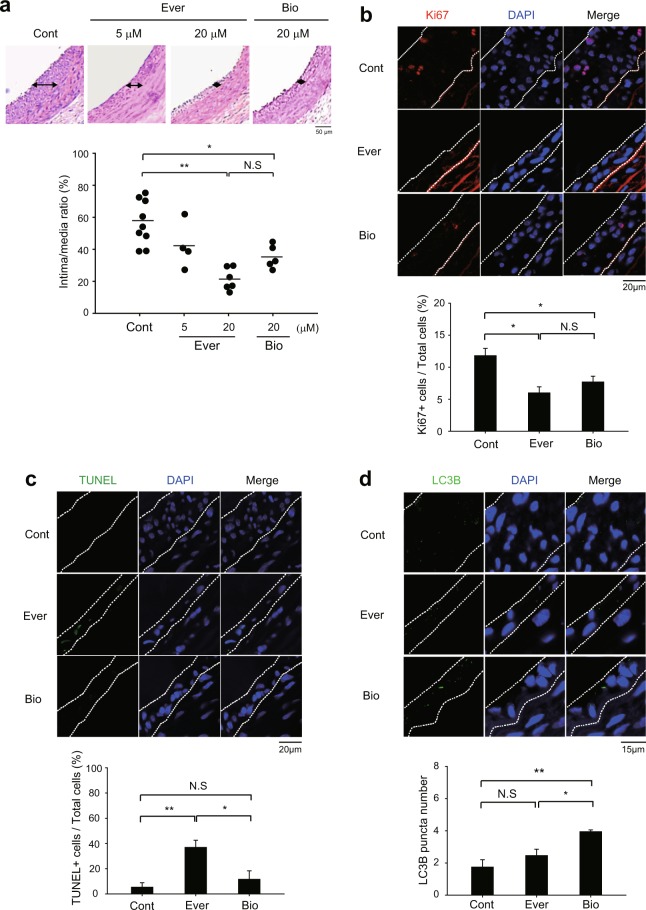
Different effects of rapamycin analogues on neointimal hyperplasia in injured carotid arteries. (**a**) Representative histological images of balloon-injured rat carotid arteries. The injured arteries were treated with control vehicle (0.1% dimethyl sulfoxide in PBS, Cont) or everolimus (5 and 20 μM in PBS, Ever) or biolimus (20 μM in PBS, Bio) for 15 min. Data in the graph are means ± SEM of intima-to-media ratio (*n* = 5–6 rats per group, ^*^P < 0.05, ^**^P < 0.001 with one-way ANOVA). N.S., not significant (**b**,**c**) Immunofluorecence staining for Ki67 (**b**) or TUNEL assay (**c**) in the tissue sections of injured carotid arteries. Data in the graph are means ± SEM of the percentages of Ki67^+^ cells (**b**) or TUNEL^+^ apoptotic cells (**c**) versus DAPI-labelled total cells in the lesion (dashed lines). DAPI indicates nuclei. (*n* = 3, ^*^P < 0.05, ^**^P < 0.01 with Student’s t-test). N.S., not significant (**d**) Immunofluorescence staining of LC3B in the tissue sections of injured carotid arteries. The number of LC3B punta was counted in the marked lesions (*dashed line*). Data in the graph show means ± SD of total number of LC3B puncta (*n* = 3, ^*^P < 0.05, ^**^P < 0.01 with Student’s t-test). N.S., not significant. Representative images are shown.

We evaluated the *in vivo* efficacy of rapalogues in a balloon injury model of rat carotid artery. As expected, everolimus and biolimus effectively inhibited the SMC hyperplasia in intimal lesion of the injured rat carotid arteries (Fig. 4a). It is noted that the inhibition of SMC hyperplasia exhibited a dose dependency proportional to the increasing concentrations of everolimus. Thus, we examined the cell proliferation and death in the tissue sections of the injured carotid arteries. Immunostaining of Ki-67 as a proliferation marker indicated a marked reduction of the neointimal VSMC proliferation in the rapalogue-treated arteries compared to untreated control arteries (Fig. 4b). Subsequently, we measured the number of apoptotic cells by a terminal deoxynucleotidyl transferase dUTP nick end labeling (TUNEL) assay. While everolimus treatment strongly induced the apoptosis in the injured arteries compared to untreated control, biolimus treatment did not induce it (Fig. 4c). This unexpected result indicated that biolimus might inhibit the SMC hyperplasia through autophagy induction. To see it, we examined the autophagosome formation in the balloon-injured carotid arteries. Immunofluorescence images of injured carotid tissue sections clearly revealed that biolimus treatment significantly increased the number of LC3B puntas compared to control and everolimus treatment (Fig. 4d).

### Biolimus prevents inflammation in porcine aorta with stent implantation

**Figure 5:**
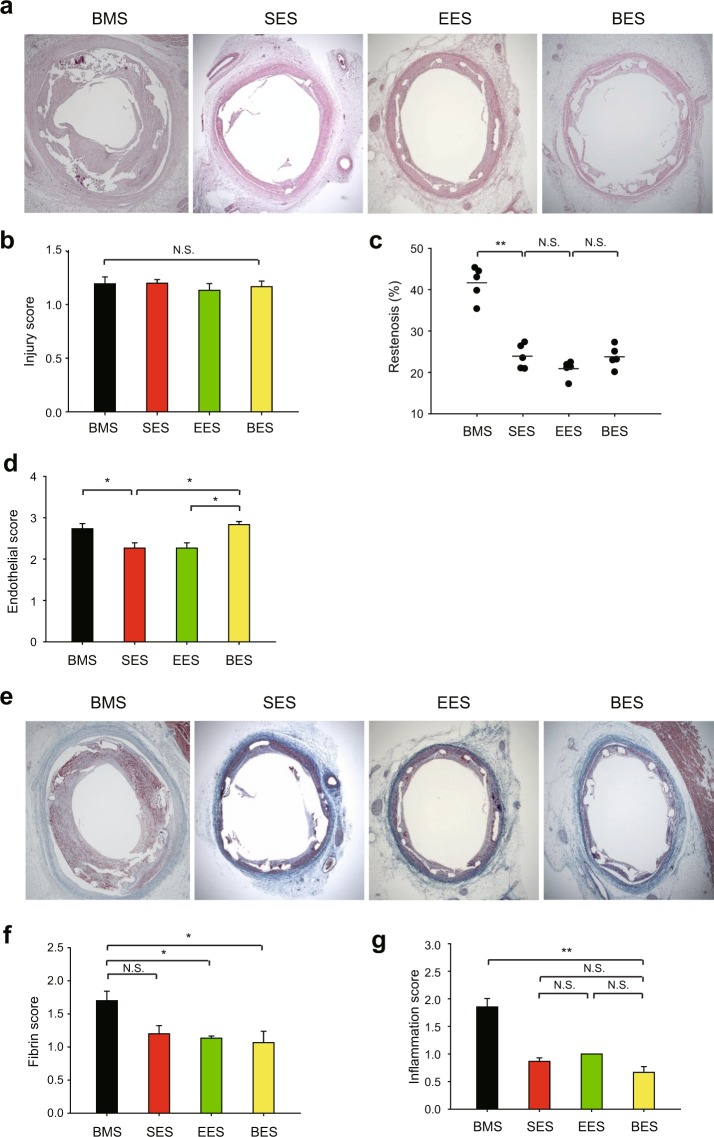
Histopathological analysis in the porcine coronary arteries with stent implantation. (**a**) Restenosis level in the cross-section of porcine coronary arteries implanted with rapalogue-coated metal stent. Representative HE images are shown. (**b**) Injury score representing similar level of vascular injury by stent implantation. (**c**) The ratio of neointimal area versus internal elastic luminal area was calculated as described in the *Materials and Methods*. Data in the graph show means ± SEM of the percent of restenosis area. (**d**) Endothelial score was measured in the tissue sections of porcine coronary arteries implanted with rapalogue-coated metal stent. (**e**,**f**) Fibrin score was measured by Carstairs’s fibrin staining in the tissue sections of porcine coronary arteries implanted with rapalogue-coated metal stent. Representative fibrin staining images are shown (**e**). (**g**) Inflammation score was measured in the tissue sections of porcine coronary arteries implanted with rapalogue-coated metal stent. Data in graphs **b**,**c**,**d**,**f**, and **g** show means ± SEM of the measured scores (*n* = 5, ^*^P < 0.05, ^**^P < 0.001 with one-way ANOVA). N.S., not significant. Bare metal stent (BMS) is used as the uncoated stent control.

To validate the differential signaling impact of rapaloues in a porcine model, we performed the percutaneous transluminal angioplasty using the metal stents coated with sirolimus and rapalogues. Drugs were coated on the metal stent by an ultrasonic spray method^25^. As a result, the drug amount per metal stent was 140 ± 8.5, 136 ± 5.9, 159 ± 7.1 μg and coating thickness was 6.5 ± 2.46, 5.8 ± 4.15, 5.9 ± 6.41 μm for sirolimus, everolimus, and biolimus, respectively. Drug release kinetics were also similar between sirolimus-eluting stent (SES) and everolimus-eluting stent (EES) during 32 days (Supplementary Figure S2). However, the drug release from the biolimus-eluting stent (BES) was significantly slow compared to other coating stents. The stents were crimped onto the balloon delivery system and sterilized with EO gas. Fifteen stents (5 SES, 5 EES and 5 BES) were successfully implanted in the porcine coronary arteries. These coronary arteries with stents were isolated to histopathological analysis (Fig. 5a). The hematoxylin and eosin staining analysis showed absolutely no difference in injury score and area stenosis among the implanted stents (Fig. 5b,c). However, biolimus-eluting stents tended to have better endothelial score (Fig. 5d) and hence slightly lower fibrin and inflammation scores than other drug-eluting stents (Fig. 5e–g). Collectively, the results suggest that autophagy-dependent VSMC arrest by biolimus might promote endothelialization and hence reduce vascular inflammation.

## Discussion

Sirolimus, also known as rapamycin, has been used in the first-generation drug-eluting stents to inhibit smooth muscle cells proliferation^4^. When metal stents are implanted to arteries, a complication of in-stent thrombosis occurs due to direct contact of circulating blood with metal stents unless the denuded endothelial cells are recovered^21,37^. Thus, advanced drug-eluting stents have been developed to alleviate the thrombosis complication^22^ or to increase the efficiency of drug delivery^23^. In general, chemical derivatization of rapamycin has been pursed at the C40 residue which does not interfere rapamycin binding to mTOR or FKBP12^38^. Although clinical studies were conducted to evaluate effects of advanced drug-eluting stents on atherosclerotic arteries^39,40^, there have been no mode of action studies to define a biological effect of such chemical derivatization on VSMC signaling.

**Figure 6:**
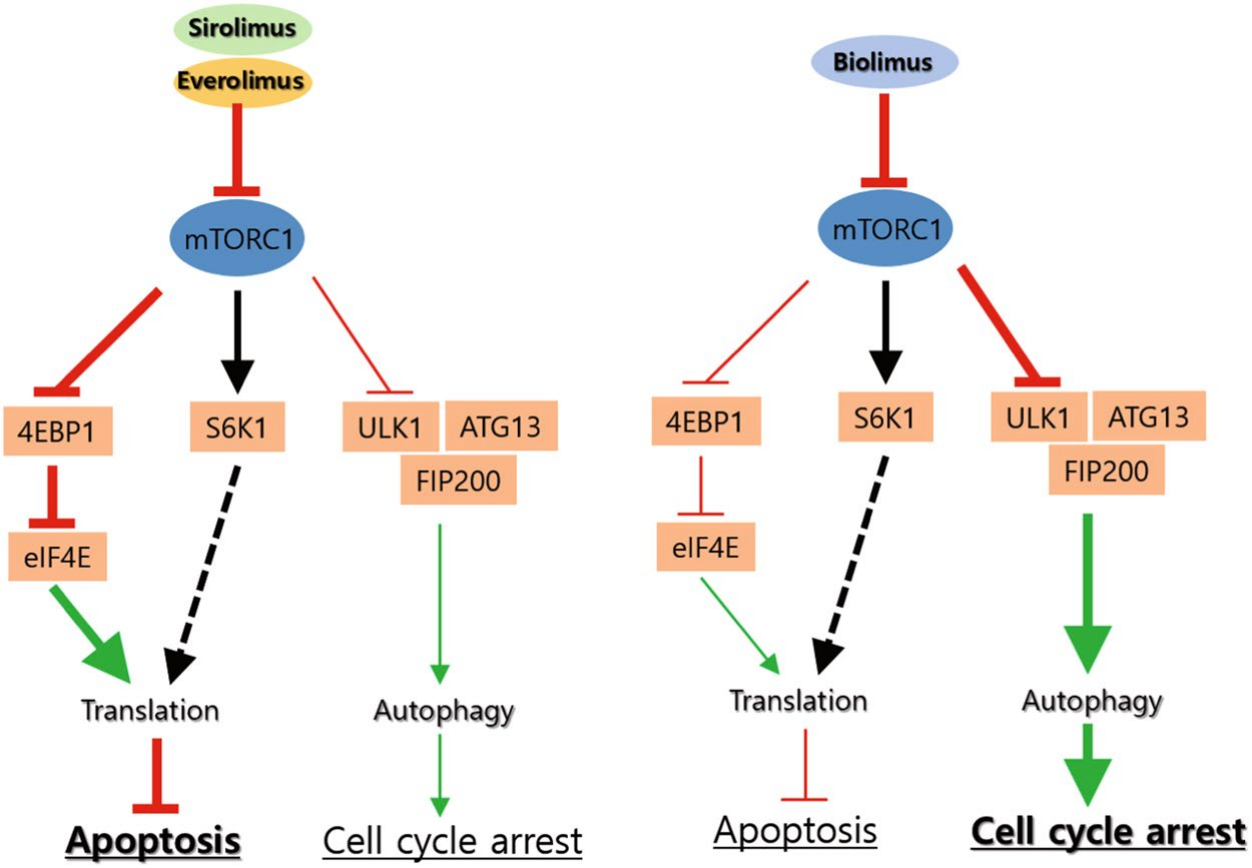
Schematic model for mode of action of rapalogues on mTORC1-dependent signaling. Sirolimus and everolimus induce a robust increase of intracellular ROS and consequently cell death, whereas biolimus preferentially relieves the inhibition of 4E-BP1 and autophagy by mTORC1 in VSMCs. Such differential mechanisms of drug action tune cellular phenotypes of VSMCs between cytotoxic and cytostatic effects.

In this study, we found that the latest rapalogue, biolimus, which has the hydrophobic C40 branch, acts on mTORC1-mediated signaling differently from the current widely used rapalogue, everolimus. The mTORC1 complex mediate activation of mRNA translation and inhibition of autophagy^33^. Our signaling pathway analyses reveal that sirolimus, everolimus, and biolimus all similarly inhibit the S6K1 activation that promotes protein translation. However, sirolimus and everolimus strongly induce intracellular ROS production and thereby cell death in VSMCs, whereas biolimus favorably activates 4E-BP1 and induces autophagy in VSMCs (Fig. 6). It is likely that sirolimus and everolimus dominantly target the signaling branch of mTORC1 complex that promotes the pentose phosphate pathway (PPP) that produces the reducing power NADPH^41^. By contrast, biolimus preferentially targets the mTORC1-dependent inhibition of the autophagy initiator ULK1 in VSMCs, which thus strengthens cell cycle arrest rather than the ROS-dependent cell death. Hence, our study provides an important message that a chemical derivatization can change the mode of drug action. It is noteworthy that the hydroxyl group of C40 residue is directed toward the kinase domain, when rapamycin binds to mTOR. This feature provided a logic for development of a dual inhibitor linking rapamycin and mTOR kinase inhibitor at C40 branch^42^. When rapalogues were fitted onto mTOR structure using an autodock modeling tool^43^, we confirmed that the C40 branches are extended toward the kinase domain of mTOR (Supplementary Figure S3). This molecular docking data implicates that the modification of C40 residue may interfere with interaction of mTORC1 with substrates. Although the C40 branch in biolimus did not directly reach the kinase domain, it may sufficiently influence overall function of large mTORC1 complex interacting with diverse downstream targets. Hence, our finding provides a critical aspect on chemical derivatization strategy for designing small-molecule inhibitors that target a large protein complex.

We also revealed that biolimus inhibits neointimal hyperplasia as efficient as other drugs, although it does not provoke cell death. Unlike sirolimus and everolimus, biolimus preferentially activates ULK1-dependent autophagy process in VSMCs. Autophagy is a dynamic recycling system for maintaining cellular homeostasis by degrading cytoplasmic materials^44^. In the process of autophagy, cytoplasmic substances and organelles are encircled by membrane to form autophagosome. Then, outer membrane of autophagosome is combined with lysosome. Internal materials are degraded by lysosomal enzymes in the autolysosome^45^. Defective autophagy is associated with various diseases including cancer, neurodegeneration, and metabolic diseases^46^. Autophagy has been broadly studied in VSMCs^35^. For example, insulin-like growth factor-1 and TNF-α inversely regulate autophagy in human VSMCs^47^. Autophagy induced by PDGF-BB promotes contractile-to-synthetic phenotype transition and increases proliferation of VSMCs by degrading contractile proteins^48^. Sonic hedgehog, another secretory factor, also induces autophagy and thus promotes VSMC proliferation^49^. By contrast, it was shown that rapamycin induces VSMC differentiation through the inhibition of mTORC1/S6K1 axis^50,51^. However, the precise regulatory action of rapalogues on VSMC autophagy remains unclear. In our study, cellular ROS and ATP levels remains unchanged after biolimus treatment, which indicates that biolimus is unlikely to induce autophagic cell death. Instead, the biolimus-induced autophagy contributes in enhancing cell cycle arrest and consequently inhibiting neointimal hyperplasia *in vivo* as effectively as do sirolimus and everolimus. Similarly, we have shown that a chemical compound targeting monoamine transporter induces autophagy in VSMCs, which sufficiently inhibits neointimal hyperplasia in the injured carotid vessels^52^. In addition, we cannot exclude a possibility that the enhanced 4E-BP1 function also contributes to cytostatic effect of biolimus because the 4E-BP1 de-phosphorylation inhibits cell proliferation^53^. Overall, our *in vitro* and *in vivo* data demonstrate that a distinct feature of biolimus selective to autophagy induction is clinically beneficial by lowering thrombotic complication.

In summary, our in-depth signaling study reveals that a minor chemical modification on rapamycin can elicit an unexpected effect in the mTORC1-mediated signaling pathways. Thus, this evidence provides an insight on medicinal chemistry for drug candidates targeting protein complexes that orchestrates diverse signaling pathways.

## Electronic supplementary material

Supplementary Information
