# Supplementary material for: A rapamycin derivative, biolimus, preferentially activates autophagy in vascular smooth muscle cells
Source: Sci Rep. 2018 Nov 8;8:16551. doi: 10.1038/s41598-018-34877-8 (PMC6224423; doi:10.1038/s41598-018-34877-8)
Supplement: Supplementary file 1 — Supplementary Information [file 41598_2018_34877_MOESM1_ESM.pdf]

A rapamycin derivative, biolimus, preferentially activates autophagy in vascular smooth muscle cells

Yerin Kim [1,*], Jun Kyu Park [3,*], Jun-Hyuk Seo [3], Hyun-Seung Ryu [3], Kyung Seob Lim [4], Myung Ho Jeong [4], Dong Hoon Kang [1,¶,#], Sang Won Kang [1,2,¶]

[1] Department of Life Science, Ewha Womans University, Seoul 03760, Republic of Korea; [2] Vasthera Co. Ltd. Seoul 03760, Republic of Korea; [3] CGBio Ltd. Jangseong 57248, Republic of Korea; [4] Cardiovascular Research Center, Chonnam National University, Gwangju 61469, Republic of Korea.

Running title: Differential VSMC signaling of rapamycin analogues

[*] These authors contributed equally to this work.

[#] Present address: Department of Asan Institute for Life Science, Asan Medical Center, College of Medicine, University of Ulsan. Seoul 05505, Republic of Korea.

[¶] Correspondence: Dong Hoon Kang, Ph.D., E-mail: ref423@hanmail.net ; Sang Won Kang, Ph.D., E-mail: kangsw@ewha.ac.kr;

a

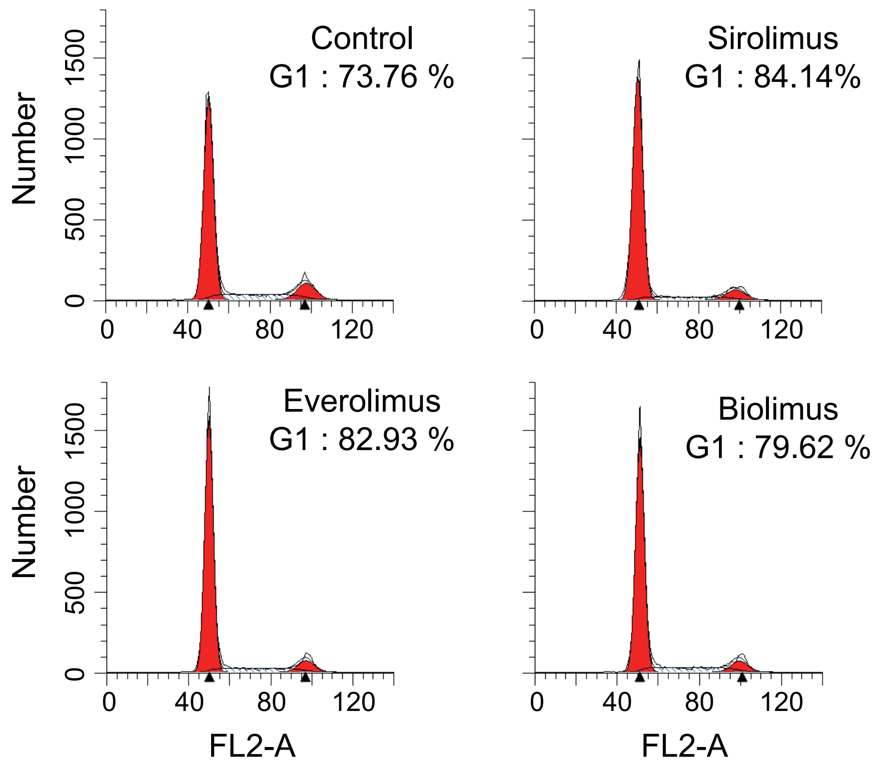

b

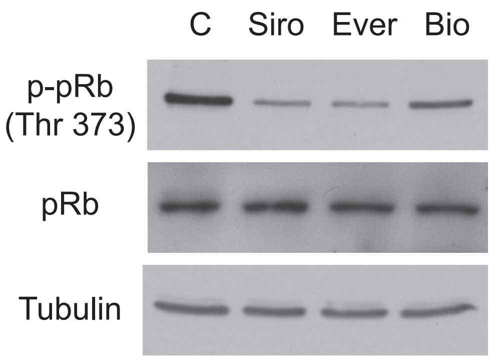

Supplementary Fig. 1 Cell cycle analysis in sirolimus- and biolimus-treated HASMCs.
a HASMCs were treated with 2 μM of sirolimus and rapalogues for 24 hr. The histogram shows PI-stained total DNA contents in cells.
b Immunoblot analysis of pRb Thr373 phosphorylation. 2 μM of sirolimus and rapalogues were treated to HASMCs for 24 hr. Immunoblot was performed using phospho-Thr 373 and total pRb antibodies. C, control; Siro, sirolimus; Ever, everolimus; Bio, biolimus.

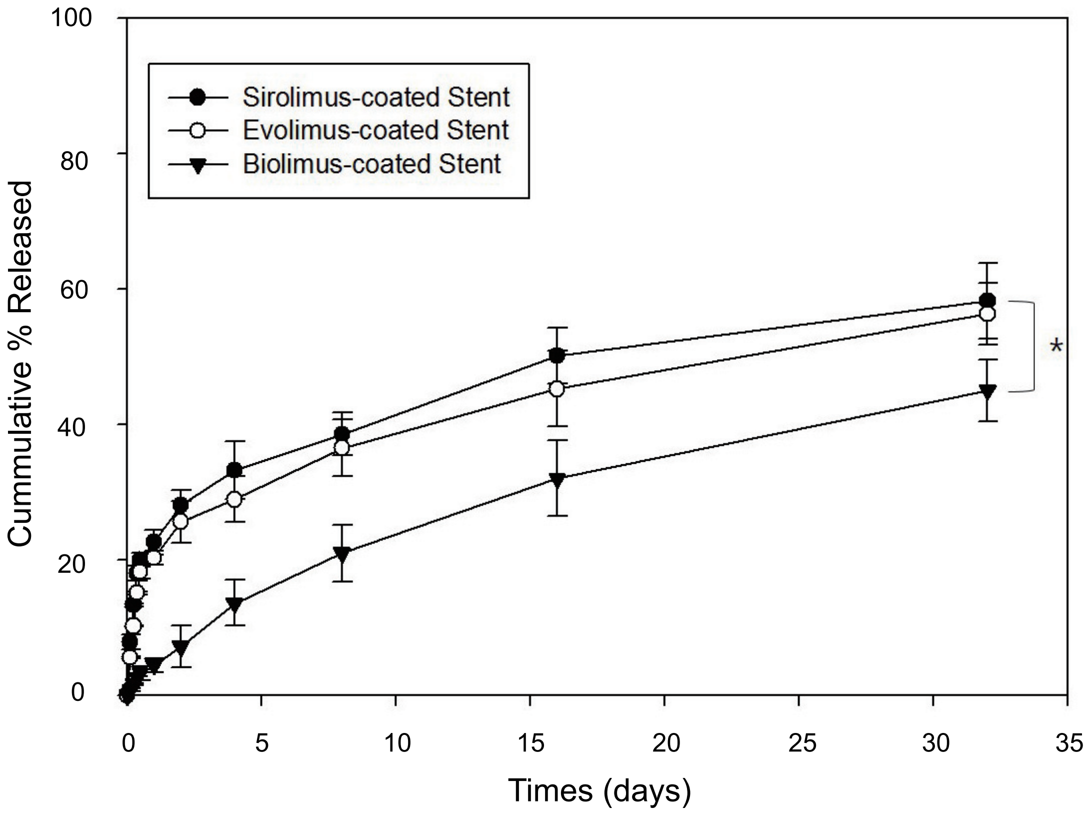

Supplementary Fig. 2. In vitro release profiles on the stent.
The drug amount and release profile on the metal stent was measured. For drug release test, coating stent were analyzed for drug release in 5 ml of PBS (pH 7.4) at 37 ºC in shaking incubator (SI-600, JEIO tech) at 125 rpm. The resulting PBS was taken at indicated time point and then was determined at 278 nm using a UV-Visible spectrophotometer (UV-1800, Shimadzu, Japan).
Total drug amount per metal stent was 140 ± 8.5 μg.

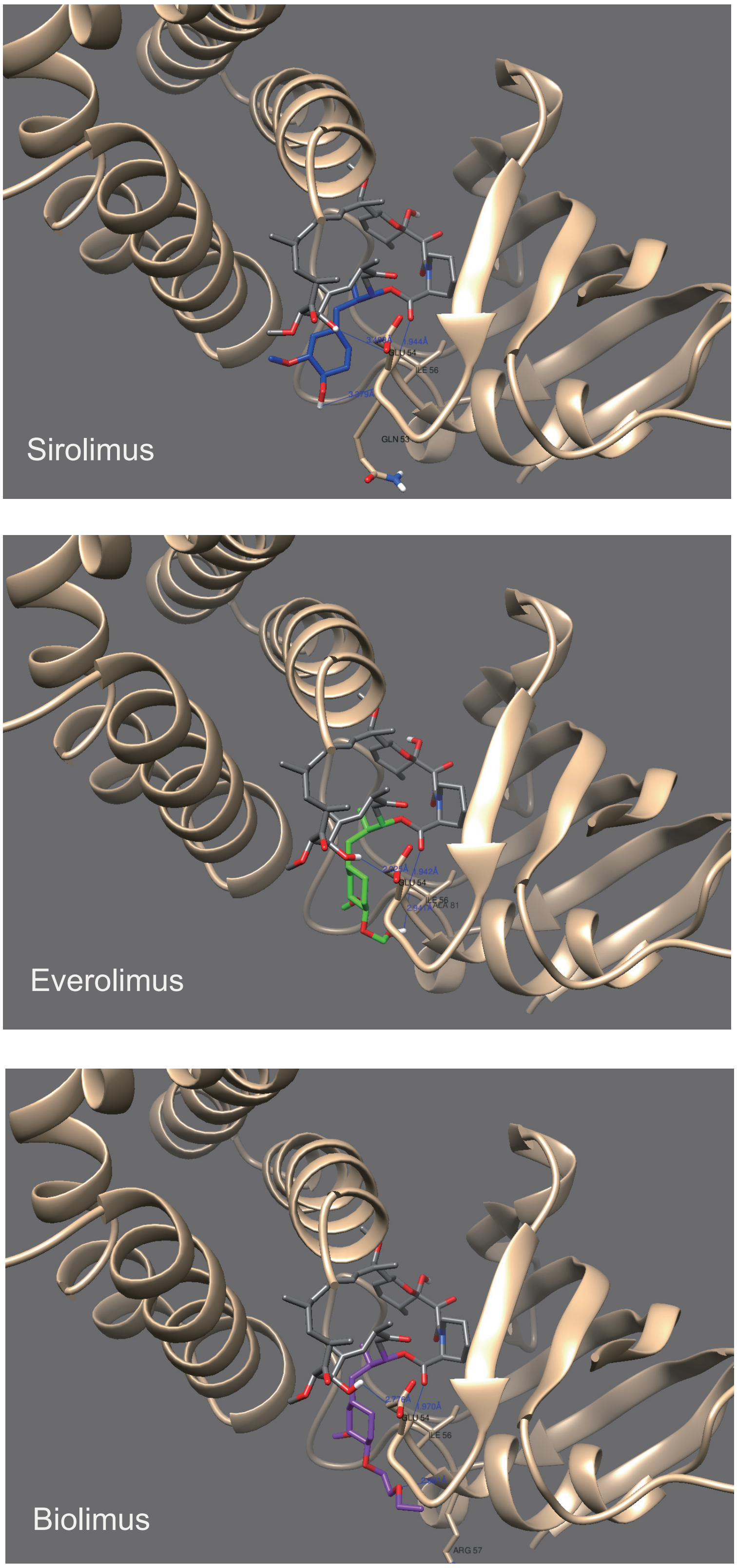

Supplementary Figure 3 Predicted conformations of rapamycin analogues bound to FRB domain of mTOR and FKBP12.

Differences in binding conformations of sirolimus, everolimus, and biolimus were analyzed using AutoDock Vina in Python Prescription 0.8. The helical structure on the left is the FRB domain of mTOR and the sheet structure on the right is the FKBP12 protein. Images show binding structures of rapamycin analogues with the largest negative values of binding score (kcal/mol). The moieties including C40 residue are shown in blue, green, and purple colors.
